# A novel encoder-decoder model based on Autoformer for air quality index prediction

**DOI:** 10.1371/journal.pone.0284293

**Published:** 2023-04-13

**Authors:** Huifang Feng, Xianghong Zhang

**Affiliations:** College of Mathematics and Statistics, Northwest Normal University, Lanzhou, China; Wuhan University of Technology, CHINA

## Abstract

Rapid economic development has led to increasingly serious air quality problems. Accurate air quality prediction can provide technical support for air pollution prevention and treatment. In this paper, we proposed a novel encoder-decoder model named as Enhanced Autoformer (EnAutoformer) to improve the air quality index (AQI) prediction. In this model, (a) The enhanced cross-correlation (ECC) is proposed for extracting the temporal dependencies in AQI time series; (b) Combining the ECC with the cross-stage feature fusion mechanism of CSPDenseNet, the core module CSP_ECC is proposed for improving the computational efficiency of the EnAutoformer. (c) The time series decomposition and dilated causal convolution added in the decoder module are exploited to extract the finer-grained features from the original AQI data and improve the performance of the proposed model for long-term prediction. The real-world air quality datasets collected from Lanzhou are used to validate the performance of our prediction model. The experimental results show that our EnAutoformer model can greatly improve the prediction accuracy compared to the baselines and can be used as a promising alternative for complex air quality prediction.

## Introduction

With the sustainable development of the economy, the environmental system on which human beings depend for survival is increasingly challenged by environmental pollution [1]. Air pollution has become one of the biggest threats to human health and life safety. The air quality index (AQI) is an important metric for quantitative evaluation of air quality conditions. It is calculated based on China Air Quality Standard (GB3095–2012) [2] for the six pollutants in the unified evaluation standard:PM_2.5_, PM_10_, CO,O_3_, SO_2_, NO_2_. According to the Technical regulation on ambient air quality index (on trial) (HJ 633–2012) issued by the Ministry of Environmental Protection of the People’s Republic of China, the AQI index is divided into 6 levels [3]. Classification standards and scope are shown in Table 1. Having a good environment is the basis of human survival and health, and various diseases have been proven to be closely related to environmental pollution. Therefore, accurate AQI prediction is important for the early warning and management of atmospheric ecology [4].

**Table 1:** AQI rating grade.

AQI range	Level	Air Quality grade	AQI stands for color
0–50	Level 1	Good	Green
51–100	Level 2	Moderate	Yellow
101 150	Level 3	Unhealthy for Sensitive Groups	Orange
151–200	Level 4	Unhealthy	Red
201–300	Level 5	Very Unhealthy	Purple
>300	Level 6	Hazardous	Maroon

In recent years, several methods have been proposed to solve the AQI prediction problem. The existing prediction methods are broadly categorized into three types, such as traditional time series, traditional machine learning and deep learning models. The prediction models based on traditional time series methods mainly include nonlinear autoregressive (NAR), autoregressive moving average (ARMA), nonlinear autoregressive moving average (NARMA), autoregressive integrated moving average (ARIMA), etc. Carlos et al. [5] applied the ARIMA model to analyze the PM_10_ concentration in high-altitude megacity by evaluating the impact of land surface cover on PM_10_ and achieved a better performance. An ARIMA model is employed to predict the air quality in New Delhi, India. The results showed that the ARIMA can capture the non-stationary of air quality and obtain the satisfactory results [6]. Erdinc et al. [7] divided the PM_10_ into three levels by utilizing the maximal overlap discrete wavelet transformation (MODWT). For each subseries obtained, the ARIMA model is used for prediction. Bhatti et al. [8] performed an analysis of mass concentration particles through correlations between air pollutants. A seasonal ARIMA (SARIMA) model was constructed and predicted future PM_2.5_. Alyousifi et al. [9] determined the transfer probability matrix of the Markov chain model by the maximum posterior method. This study provided an important reference for scientific prevention and control of air pollution.

Compared with traditional statistical methods, machine learning does not need to make any assumptions about the data. Meanwhile, it could achieve accurate prediction results by using cross-validation methods. The traditional machine learning models include logistic regression (LR), decision tree (DT), support vector regression (SVR), random forest (RF), Naive Bayes (NB), K-Nearest Neighbors (KNN), Pugliese et al [10]. Liu et al. [11] used the support vector machine (SVM) model optimized by different algorithms to assist in predicting the PM_2.5_ levels and achieved good prediction accuracy. However, when the training sample is large, the memory and implementation of the matrix is a big challenge for SVM algorithms. Xia [12] used RF and cluster analysis methods to investigate the air quality distribution of Changsha and further used ARMA model for prediction. Choubin et al. [13] used multiple machine learning models, which included the bagged CART, mixture discriminant analysis and random forest, to predict the hazard of particulate matter (PM). Liu et al. proposed a fusion model PCR-SVR-ARMA to predict air pollutants that incorporating principal component regression (PCR), SVR, and ARMA [14]. Rajat et al. [15] employed four supervised machine learning methods, which included DT, RF, NB and KNN, for prediction of AQI. The results showed that the DT gave the best performance among all the models. Ma et al. [16] used gradient boosting algorithm to predict the PM_2.5_ in the Jing-Jin-Ji area. Their results showed that the model could more accurately predict the next day’s PM_2.5_ based on the data of the previous 5 days. However, when the data is large, the algorithm will consume a lot of computing time. Ke et al. [17] developed an air quality prediction system based on machine learning for predicting six common pollutants and pollution levels. The seven datasets collected from the typical central cities in China are implemented. Experiment results show that the proposed model can achieve reliable short-term air quality prediction. Traditional machine learning methods focus on short-term traffic flow prediction and can achieve good prediction accuracy. However, traditional machine learning models have simple architectures and limited parameters, and cannot tap into the deeper, implied spatio-temporal correlations in big data, so they have limited capability for medium- and long-term prediction.

In recent years, deep learning (DL) has developed rapidly and has become the newest trends of scientific research. The deep learning models include multi-layer perceptrons (MLP), convolutional neural networks (CNN), long-short-term memory (LSTM), Gated Recurrent Unit (GRU), etc. Compared to traditional machine learning, deep learning methods use deep neural networks to perform more sophisticated processing on the model, resulting in a more powerful feature mining capability. Deep learning has been applied to the fields of meteorology and environmental science. The conditional local convolution recurrent network (CLCRN) [18] were employed for modeling the meteorological flows of local patterns on the whole sphere. Four hour-wise weather datasets including temperature, cloud cover, humidity and surface wind component were used for performance evaluation. Lv et al. [19, 20] employed the deep learning for wind speed prediction. The hybrid deep learning models, which combined with feature selection, time series decomposition, and multi-objective parameter optimization, were proposed to predict the wind speed. A location-refining neural network combined the optical flow-based methods with the deep learning-based methods was proposed for the heavy rainfall prediction [21]. The LSTM-based prediction model was employed to estimate sea surface temperatures and predict high water temperature [22].

Air quality prediction involves a variety of factors, including pollutant concentrations and meteorology, and in particular, changes in meteorological conditions can lead to large fluctuations in pollutant concentrations, thus making prediction more difficult. Deep learning models can capture these complex features of air quality. Agarwal et al. [23] used Artifificial Neural Networks (ANN) to predict the pollutant concentration (PM_10_, PM_2.5_, NO_2_, O_3_) with the data colledcted from Delhi. The model dynamically adjusts prediction with equipped real-time corrections to improve forecast quality. Zhou et al. [24] constructed a deep multi-output LSTM (DM-LSTM) model through deep learning algorithms and predicted the concentration of relevant pollutants in Taipei, Taiwan, which significantly improved the accuracy and stability of air quality forecasting. Aggarwal et al. [25] proposed a hybrid model (P-LSTM) based on LSTM and particle swarm optimization(PSO) to predict the air quality collected from 15 locations in India. Experimental results show that PSO can optimize LSTM network parameters and improve prediction performance. Yan et al. [26] constructed multiple AQI models to predict future data by learning the change regularity of air quality data. The comparison found that LSTM has the best performance. Liu et al. [27] proposed an attention-based air quality predictor (AAQP) to forecast the air quality index of Beijing in the future. Dun et al. [28] proposed a DGC-MTCN model, which combined dynamic graph convolutional network (DGC) and multi-channel temporal convolutional network (MTCN) to predict the PM_2.5_ in Beijing and Fushun and achieved good prediction accuracy.

In 2017, the Google team proposed a sequence-to-sequence model with attention mechanisms [29] for machine translation tasks, which changed the previous way of recursive transmission of sequence information and instead processed sequence information as a whole. In 2019, researchers took full advantage of the Transformer and improved the calculation of attention based on the Transformer to accommodate time series data [30]. In recent years, transformer-based models have achieved excellent results in capturing dependencies over long distances, such as Sparse Transformer [31], Reformer [32], Informer [33], Autoformer [34], etc. Various types of transformer-based models are being applied to time series prediction [31–35]. Taking the advantage of hybrid deep learning techniques, this study proposes an AQI prediction approach based on the combination of enhanced feature extraction, cross-stage feature fusion mechanism, data decomposition method, and deep learning model. The main contributions are listed as follow:

- We propose a novel encoder-decoder model named as Enhanced Autoformer (EnAutoformer), which is an improvement of Autoformer, to predict the AQI. The EnAutoformer model consists of three major modules: feature extraction and fusion module (CSP_ECC), data decomposition module, and dilated causal convolution module.
- An enhanced cross-correlation (ECC) is proposed for extracting the temporal-dependent features in AQI time series.
- A CSP_ECC mechanism is designed by integrating the cross-stage feature fusion mechanism of CSPDenseNet and ECC mechanism. CSP_ECC is not only able to extract the temporal dependencies in original time series, but also to improve the computational efficiency.
- To further obtain the finer-grained information, the series decomposition is developed to concurrently extract the frequency-domain features including seasonality and trend from original time series.
- A dilated causal convolution network is employed to capture long-range dependencies of original time series, further enhanced the long-term predictive ability of the EnAutoformer model.
- To evaluate the effectiveness of EnAutoformer, the experiments with five real-world air quality datasets collected from different regions of Lanzhou are implemented. Compared with the baselines, experimental results show that our proposed model EnAutoformer achieves significant predictive performance.

The organization of this paper is as follows. Section 2 introduces the methodology and proposes the prediction model. Section 3 describes datasets, baseline models, the experimental settings, and discusses the results of the experiments. The conclusions are given in Section 4.

## Methodology

### Enhanced cross-correlation

**Fig 1:**
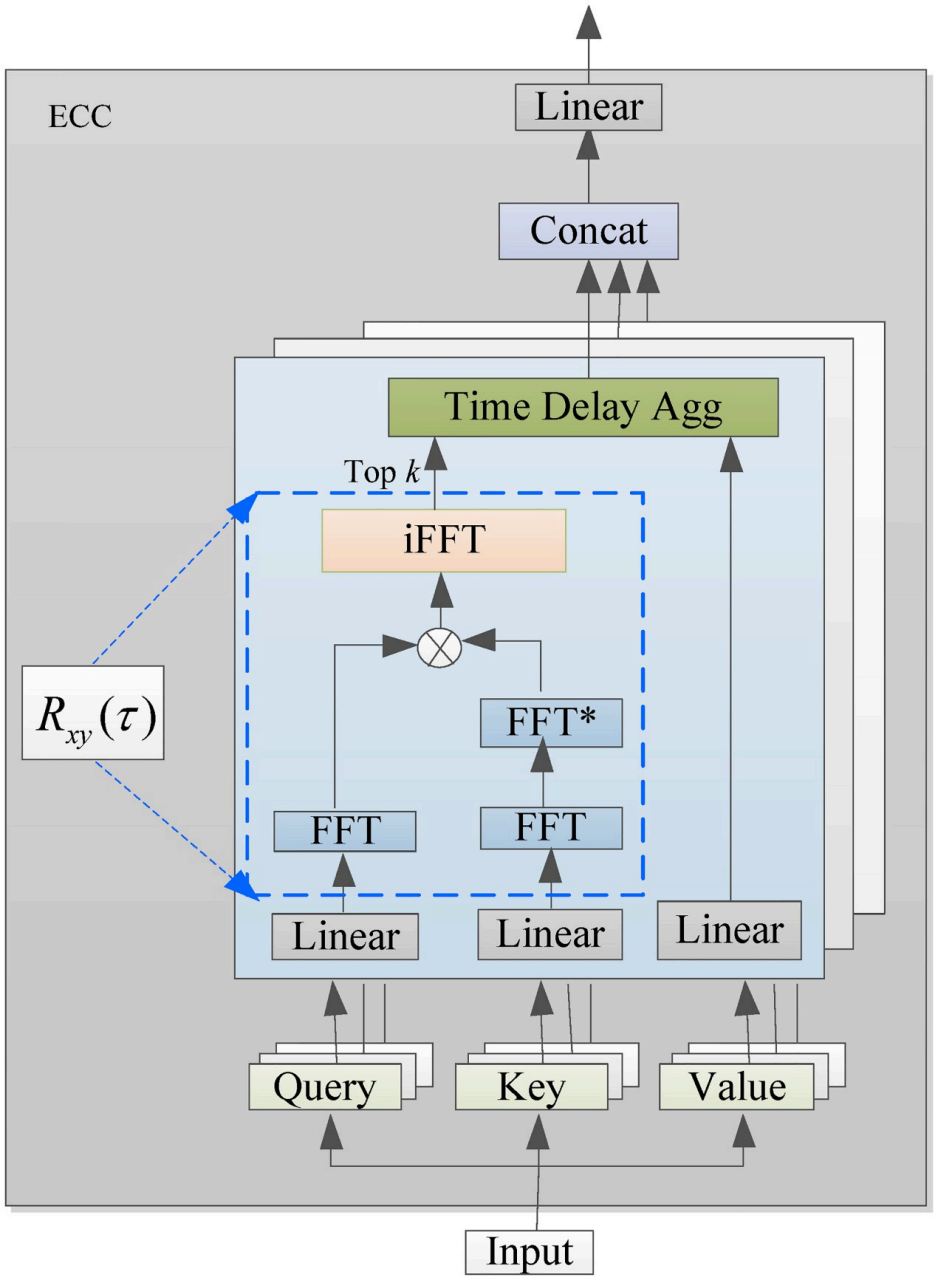
The structure of the ECC.

Enhanced Cross-correlation (ECC) consists of two core modules, a cross-correlation module to detect time-shifted correlations between time series, and a time-delayed aggregation module to aggregate the strongly correlated ones. The structure of the ECC is shown in Fig 1.

Cross-correlation is often used to measure the similarity of a time series *x*(*t*) and shifted (lagged) copies of a time series *y*(*t*) as a function of the lag *τ*. The lag when the cross-correlation value reaches its maximum is the lag when the two time series are best correlated. The cross-correlation function *R*_*xy*_(*τ*) at lag *τ* is defined as:

$$\begin{array}{c} R_{xy}\left(\tau \right)=\sum_{t=-\infty }^{+\infty }x\left(t\right)y\left(t+\tau \right). \end{array}$$ (1)

Fast Fourier Transform (FFT) is an indirect method to calculate the cross-correlation function and the calculation process is shown in the blue block in Fig 1. The FFT and inverse FFT of the discrete signal *x*(*t*) can be calculated as:

$$\begin{array}{c} X\left(k\right)=\sum_{n=0}^{N-1}x\left(n\right)e^{\left(-j2\pi kn/N\right)},k=0,1,\cdots ,N-1, \end{array}$$ (2)

$$\begin{array}{c} x\left(n\right)=\frac{1}{N}\sum_{n=0}^{N-1}X\left(k\right)e^{\left(+j2\pi kn/N\right)},k=0,1,\cdots ,N-1, \end{array}$$ (3)

The cross-correlation function can be computed using FFT algorithm based on the convolution theorem, which can be expressed as follows:

$$\begin{array}{c} R_{xy}\left(\tau \right)=\text{iFFT}\left({\text{FFT}}_{x}\cdot {\text{FFT}}_{y}^{*}\right), \end{array}$$ (4)

where FFT_*x*_ and FFT_*y*_ are the Fourier transform of *x*(*t*) and *y*(*t*), respectively, * means complex conjugation and iFFT(⋅) stands for the inverse FFT. Compared with the direct calculation method, the indirect calculation method can reduce the time complexity of cross-correlation from *O*(*N*^2^) to *O*(*Nlog*(*N*)), so it has obvious superiority in the analysis of large data sample size.

Auto-correlation describes the degree of every correlation between two couples of the time series delayed by the lag. Similar to the cross-correlation function, the definition and calculation of the auto-correlation function are respectively as follows:

$$\begin{array}{c} R_{xx}\left(\tau \right)=\text{iFFT}\left({\text{FFT}}_{x}\cdot {\text{FFT}}_{x}^{*}\right). \end{array}$$ (5)

The time delay aggregation is an alignment aggregation of time-shift time series with top *k* correlation ranking, which is selected by the cross-correlation function. The time delay aggregation (TDA) is expressed as follows [34]:

$$\begin{array}{c} \tau_{1},\tau_{2},\cdots ,\tau_{k}={\text{arg}}_{\tau \in \{1,2,\cdots ,L\}}\text{Topk}\left(R_{Q,K}\left(\tau \right)\right), \end{array}$$ (6)

$$\begin{array}{c} {\hat{R}}_{Q,K}\left(\tau_{1}\right),{\hat{R}}_{Q,K}\left(\tau_{2}\right),\cdots ,{\hat{R}}_{Q,K}\left(\tau_{k}\right)=\text{SoftMax}\left(R_{Q,K}\left(\tau_{1}\right),R_{Q,K}\left(\tau_{2}\right),\cdots ,R_{Q,K}\left(\tau_{k}\right)\right), \end{array}$$ (7)

$$\begin{array}{c} \text{TDA}\left(Q,K,V\right)=\sum_{i=1}^{k}\text{Roll}\left(V,\tau_{i}\right){\hat{R}}_{Q,K}\left(\tau_{i}\right), \end{array}$$ (8)

where Topk(⋅) is the function used to select the top *k* time series with the strongest correlation. SoftMax(⋅) is the normalized exponential function. Roll(⋅) is a function that shifts the time series according to the offset.

For the multi-head mechanism,

$$\begin{array}{c} \text{MultiHeadTDA}\left(Q,K,V\right)=W_{\text{output}}\text{Concat}\left({\text{head}}_{1},{\text{head}}_{2},\cdots ,{\text{head}}_{h}\right), \end{array}$$ (9)

where head_*i*_ = TDA(*Q*_*i*_, *K*_*i*_, *V*_*i*_), Concat(⋅) is the concatenation function.

### CSP_ECC

**Fig 2:**
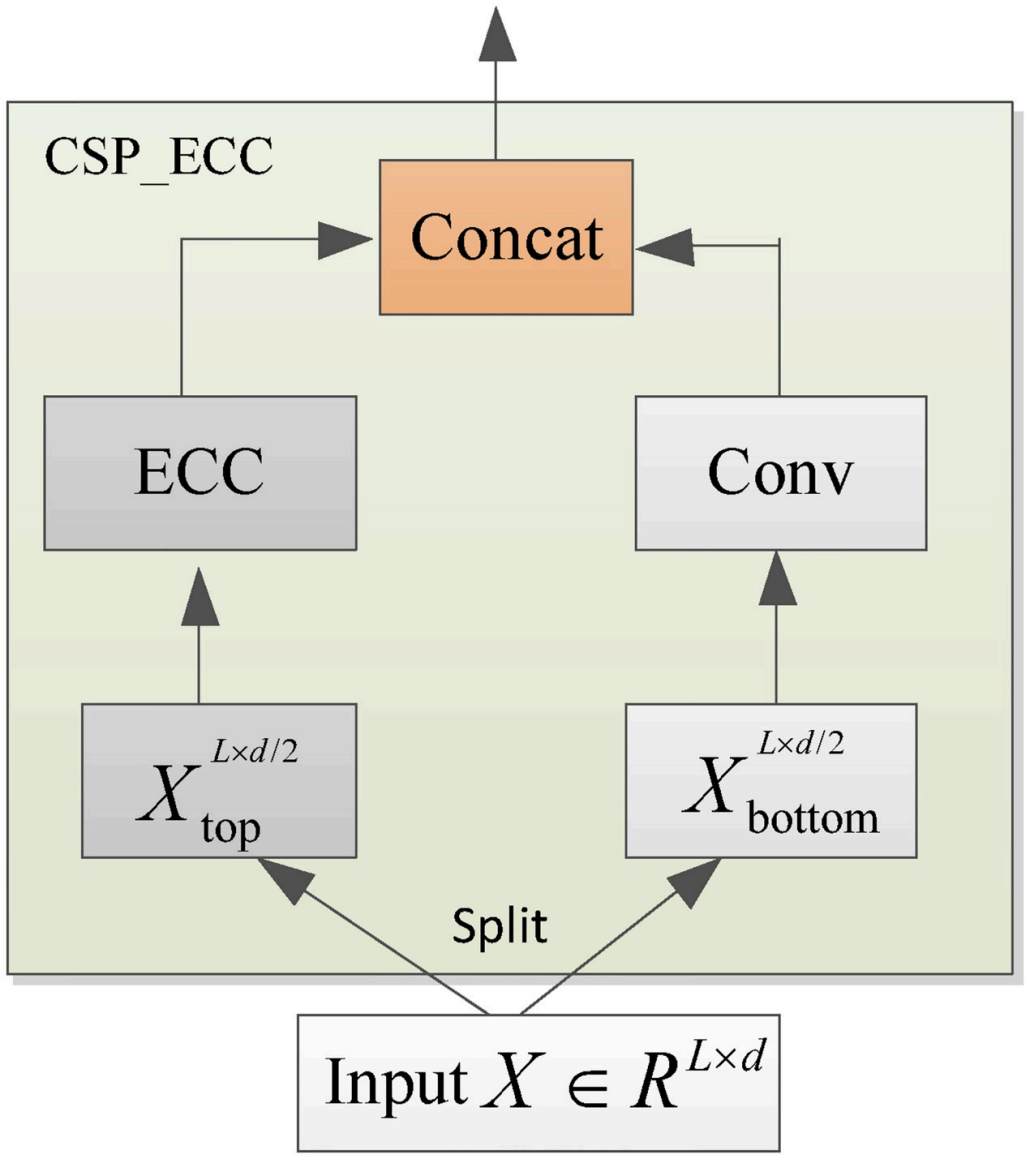
The structure of the CSP_ECC.

Inspired by CSPNet [36], CSPAttention [35], and auto-correlation mechanism [34], a cross stage partial based on enhanced cross-correlation (CSP_ECC) is proposed to capture the inherent features of AQI time series and solve the problem of high computational complexity. The structure of CSP_ECC is shown in Fig 2. The CSP_ECC consists of two blocks, one of which is an ECC block and the other is a 1 × 1 convolutional layer. CSP_ECC reduces the time complexity by reducing the input dimension [35]. We split the input *X* ∈ *R*^*L*×*d*^ in two parts $X=[X_{\text{bottom}}^{L\times d/2},X_{\text{top}}^{L\times d/2}]$, where *L* is the input length and *d* is the input dimension. $X_{\text{top}}^{L\times d/2}$ is the input of the ECC block, $X_{\text{bottom}}^{L\times d/2}$ is the input of the 1 × 1 convolution block. The outputs of two blocks are concatenated through dimension as the output of the CSP_ECC.

### Dilated causal convolution(DCC)

A dilated causal convolutional network is a multilayer convolutional neural network that can be expanded in time-domain [37]. It is employed to process long-range dependent sequences by using a non-recursive method. Dilated convolution allows the model to increase the perceptual field exponentially with fewer layers and maintain computational efficiency.

**Fig 3:**
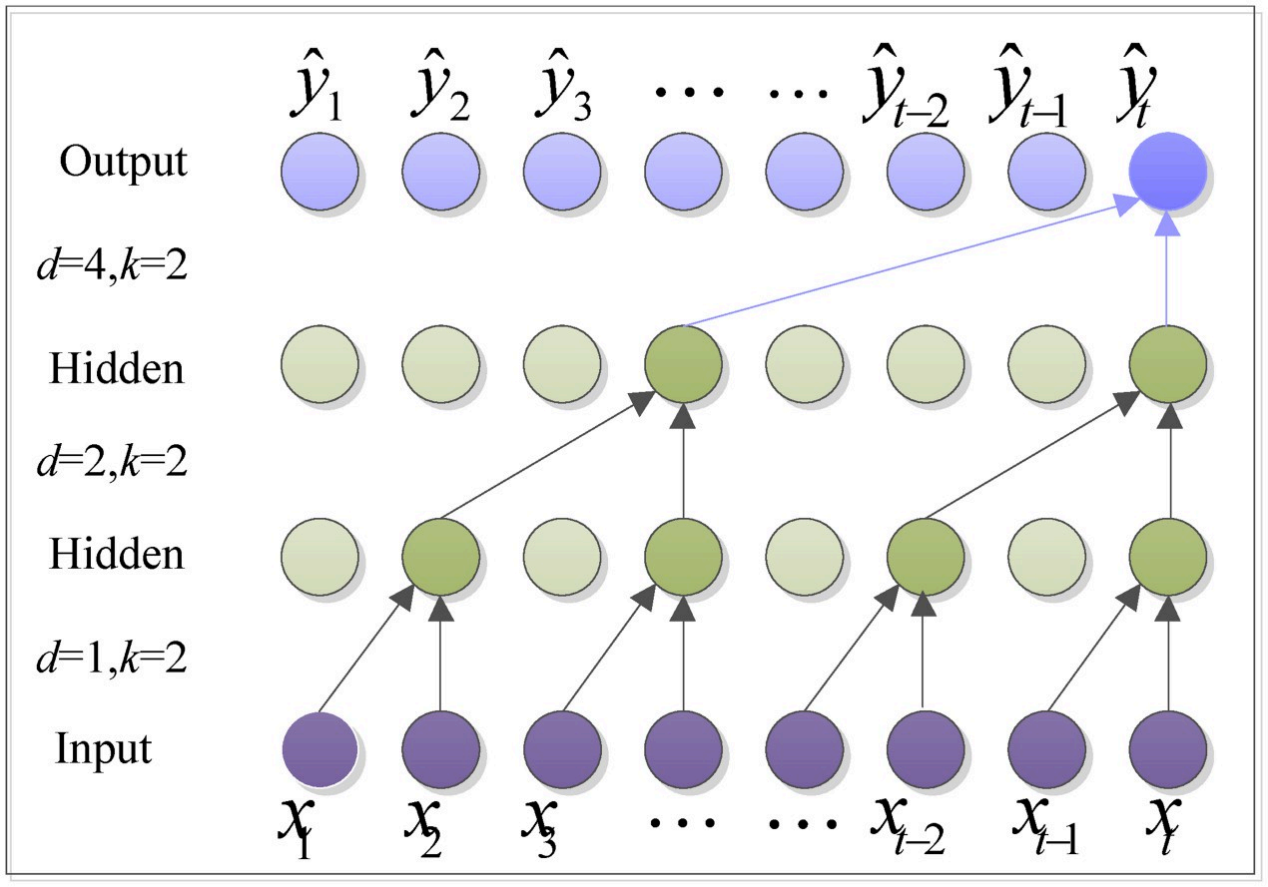
The structure of the DCC.

Given an input sequence *X* = {*x*_1_, *x*_2_, ⋯, *x*_*N*_} and the filter *F* = {*f*_1_, *f*_2_, ⋯, *f*_*k*_}. The dilation causal convolution on element *x*_*t*_ of the input *X* is defined as:

$$\begin{array}{c} \left(X{*}_{d}F\right)\left(x_{t}\right)=\sum_{i=0}^{k-1}f_{i}x_{t-d\times i}, \end{array}$$ (10)

where *_d_ denotes the dilated convolution operator, *d* is the dilation factor, and *k* is the filter size. As the depth of the model increases, the dilation factor *d* increases exponentially, i.e. *d* = 2^*l*^ at layer *l*. A dilated causal convolution with *d* = 1, 2, 4 and size *k* = 2 is shown in Fig 3.

### Time series decomposition

Time series decomposition is a very useful method that transforms a time series into multiple subseries representing different characteristics. The characteristics, trends and development patterns of variable changes are extracted from the time series to make effective prediction. There are various decomposition methods for time series. The classical seasonal decomposition is one of the time series decomposition methods. The classical seasonal decomposition method works by applying an additive or multiplicative model to divide a time series into three components: seasonality, trend and noise. In this paper, we perform the time series decomposition using a simplified additive model that decomposes the time series into trend and seasonality. The trend component is obtained by moving average of the time series. Removing the calculated trend from the time series will produce a new time series called seasonality.

### Our proposed model: EnAutoformer

**Fig 4:**
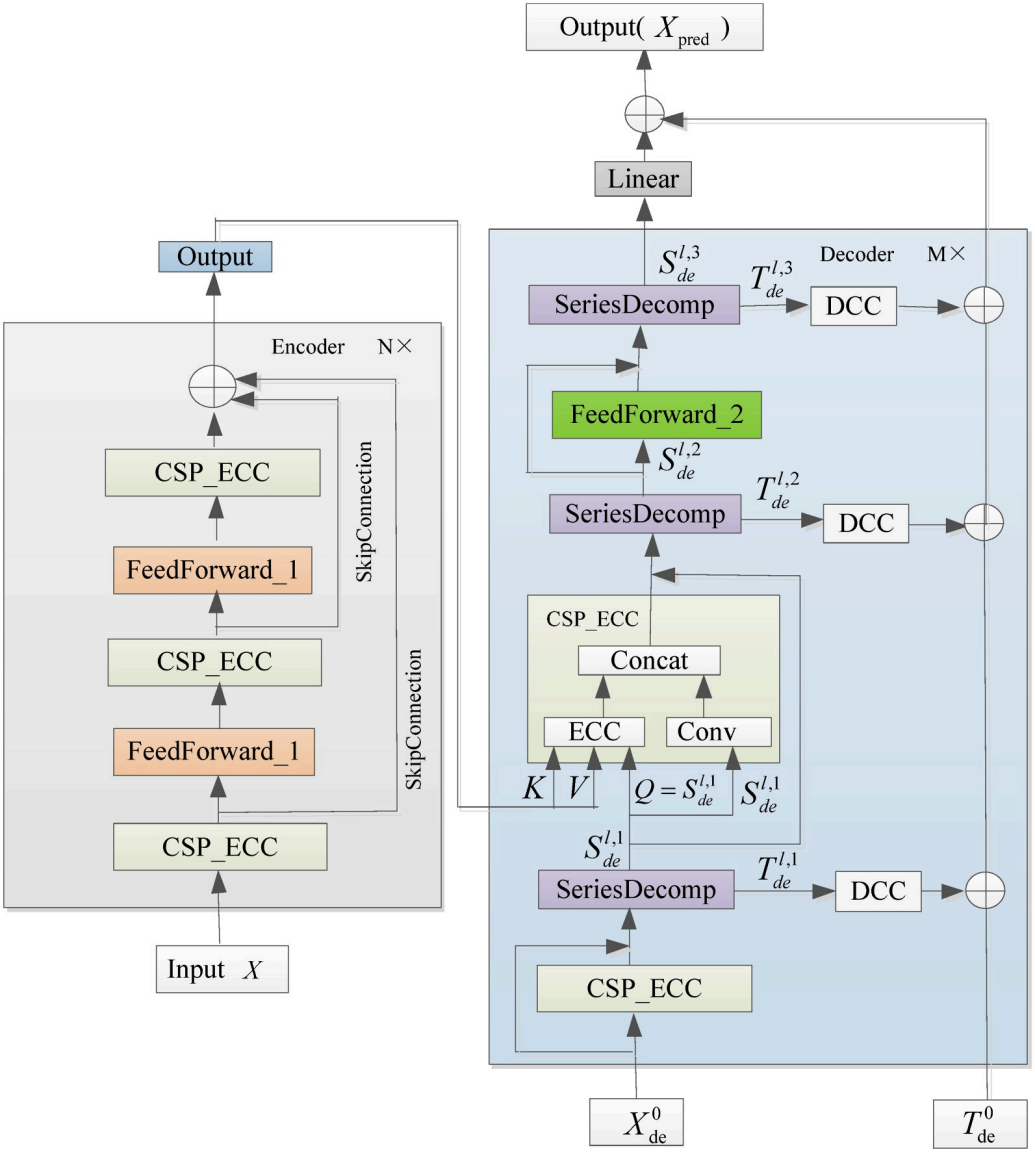
The overall architecture of the EnAutoformer for AQI prediction.

We propose a novel encoder-decoder model named as Enhanced Autoformer (EnAutoformer) for AQI prediction. The structure is represented in Fig 4. The Encoder is stacked by identical encoder layers. Each encoder layer contains three CSP_ECC blocks and two FeedForward_1 blocks.

**Fig 5:**
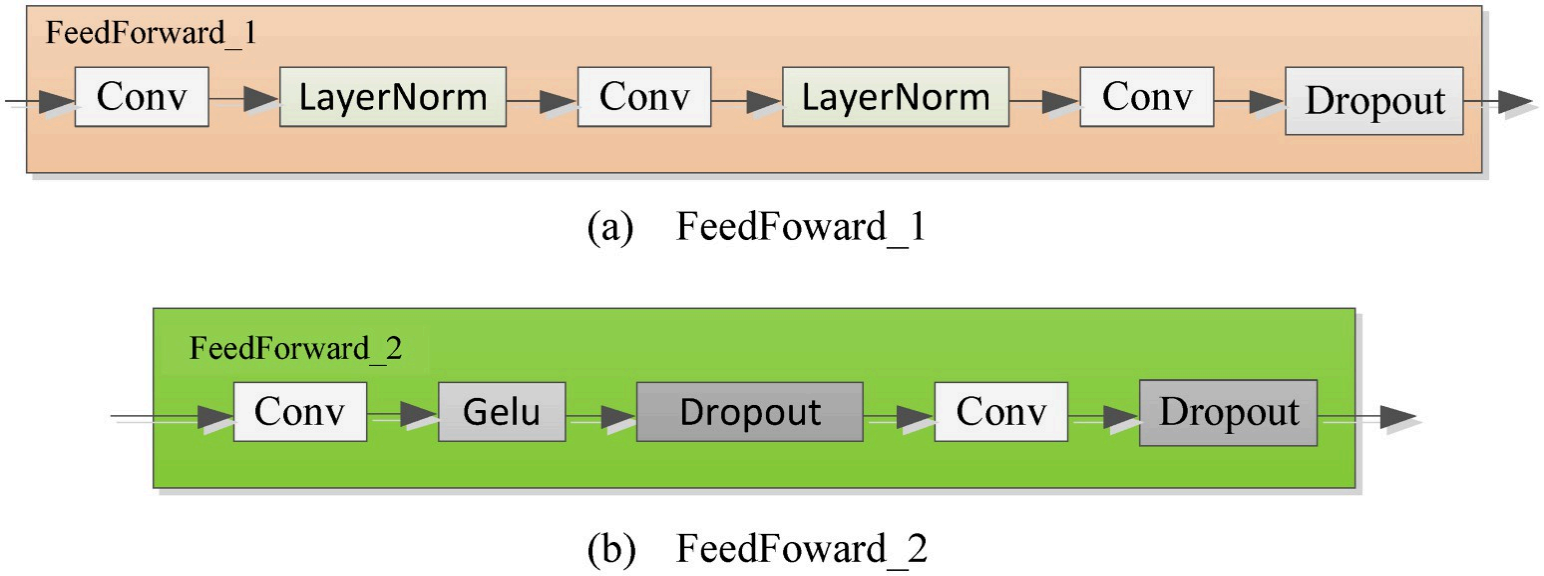
The structure of FeedForward.

The *l*-th encoder layer can be summarized as $X_{\text{en}}^{l}=\text{Encoder}(X_{\text{en}}^{l-1})$, where *l* ∈ {1, 2, ⋯, *N*} and $X_{\text{en}}^{0}$ denote the initial historical series that has been embedded with temporal information. The specific details are as follows:

$$\begin{array}{c} X_{\text{en}}^{l,1}=\text{CSP\_ECC}(X_{\text{en}}^{l-1}), \end{array}$$ (11)

$$\begin{array}{c} X_{\text{en}}^{l,2}=\text{CSP\_ECC}(\text{FeedForward}\_1(X_{\text{en}}^{l,1})), \end{array}$$ (12)

$$\begin{array}{c} X_{\text{en}}^{l,3}=\text{CSP\_ECC}(\text{FeedForward}\_1(X_{\text{en}}^{l,2})), \end{array}$$ (13)

$$\begin{array}{c} X_{\text{en}}^{l}=X_{\text{en}}^{l,1}+X_{\text{en}}^{l,2}+X_{\text{en}}^{l,3}, \end{array}$$ (14)

where $X_{\text{en}}^{l,i},i\in \left\{1,2,3\right\}$ represents the output after the *i*-th CSP_ECC in the *l*-th encoder layer. FeedForward_1(⋅) is a simple feed-forward neural network consisting of an input layer, six hidden layers and an output layer. The FeedForward_1 structure is shown in Fig 5(a).

A decoder block consists of three parts, namely CSP_ECC, SeriesDecomp and FeedForward_2. Supposing the decoder includes *M* decoder layers. The *l*-th decoder layer can be described as $X_{\text{de}}^{l},T_{\text{de}}^{l}=\text{Decoder}(X_{\text{de}}^{l-1},T_{\text{de}}^{l-1})$, where *l* = 1, 2, ⋯, *M*. The Decoder(⋅) is formalized as:

$$\begin{array}{c} S_{\text{de}}^{l,1},T_{\text{de}}^{l,1}=\text{SeriesDecomp}(\text{CSP\_ECC}\left(X_{\text{de}}^{l-1}\right)+X_{\text{de}}^{l-1}), \end{array}$$ (15)

$$\begin{array}{c} S_{\text{de}}^{l,2},T_{\text{de}}^{l,2}=\text{SeriesDecomp}(\text{CSP\_ECC}\left(S_{\text{de}}^{l,1},X_{\text{de}}^{N}\right)+S_{\text{de}}^{l,1}), \end{array}$$ (16)

$$\begin{array}{c} S_{\text{de}}^{l,3},T_{\text{de}}^{l,3}=\text{SeriesDecomp}(\text{FeedForward}\_2\left(S_{\text{de}}^{l,2}\right)+S_{\text{de}}^{l,2}), \end{array}$$ (17)

$$\begin{array}{c} T_{\text{de}}^{l}=T_{\text{de}}^{l-1}+\text{DCC}\left(T_{\text{de}}^{l,1}\right)+\text{DCC}\left(T_{\text{de}}^{l,2}\right)+\text{DCC}\left(T_{\text{de}}^{l,3}\right), \end{array}$$ (18)

$$\begin{array}{c} X_{\text{de}}^{l}=S_{\text{de}}^{l,3}. \end{array}$$ (19)

where $S_{\text{de}}^{l,i},T_{\text{de}}^{l,i},i=1,2,3$ represent the seasonality and trend after the *i*-th time series decomposition block in the *l*-th layer respectively. FeedForward_2(⋅) is a simple feed-forward neural network, and its structure is shown in Fig 5(b).

The final prediction *X*_pred_ is the sum of the refined decomposed sequence:

$$\begin{array}{c} X_{\text{pred}}=\text{Linear}\left(X_{\text{de}}^{M}\right)+T_{\text{de}}^{M}. \end{array}$$ (20)

The main steps of the prediction model and its pseudo-code are shown in Algorithm 1.

**Algorithm 1** Pseudo-code for the main prediction steps based on EnAutoformer

**Input**:

Raw time series *X*_Raw_; Input length *I*; Predict length *O*; Data dimension *d*

Encoder layers number *N*; Decoder layers number *M*

**Output**:

Prediction *X*_pred_

1: *X* = Preprocessing(*X*_Raw_)

2: Initialization $X_{\text{en}}^{0}\leftarrow X$

3: **for** l = 1:*N* **do**

4:  $X_{\text{en}}^{l,1}=\text{CSP\_ECC}\left(X_{\text{en}}^{l-1}\right)$

5:  $X_{\text{en}}^{l,2}=\text{CSP\_ECC}\left(\text{FeedForward}\_1\left(X_{\text{en}}^{l,1}\right)\right)$

6:  $X_{\text{en}}^{l,3}=\text{CSP\_ECC}\left(\text{FeedForward}\_1\left(X_{\text{en}}^{l,2}\right)\right)$

7:  $X_{\text{en}}^{l}=X_{\text{en}}^{l,1}+X_{\text{en}}^{l,2}+X_{\text{en}}^{l,3}$

8: **end for**

9: *X*_de_, *T*_de_ = SeriesDecomp(*X*_I/2:I_)

10: *X*_0_, *X*_mean_ = Zeros([*O*, *d*]), Repeat(mean(*X*_*I*/2: *I*_, dim = 0), dim = 0)

11: $X_{\text{de}}^{0},T_{\text{de}}^{0}=\text{Concat}\left(X_{\text{de}},X_{0}\right),\text{Concat}\left(T_{\text{de}},X_{\text{mean}}\right)$

12: **for** l = 1:*M* **do**

13:  $S_{\text{de}}^{l,1},T_{\text{de}}^{l,1}=\text{SeriesDecomp}\left(\text{CSP\_ECC}\left(X_{\text{de}}^{l-1}\right)+X_{\text{de}}^{l-1}\right)$

14:  $S_{\text{de}}^{l,2},T_{\text{de}}^{l,2}=\text{SeriesDecomp}\left(\text{CSP\_ECC}\left(S_{\text{de}}^{l,1},X_{\text{de}}^{N}\right)+S_{\text{de}}^{l,1}\right)$

15:  $S_{\text{de}}^{l,3},T_{\text{de}}^{l,3}=\text{SeriesDecomp}\left(\text{FeedForward}\_2\left(S_{\text{de}}^{l,2}\right)+S_{\text{de}}^{l,2}\right)$

16:  $T_{\text{de}}^{l}=T_{\text{de}}^{l-1}+\text{DCC}\left(T_{\text{de}}^{l,1}\right)+\text{DCC}\left(T_{\text{de}}^{l,2}\right)+\text{DCC}\left(T_{\text{de}}^{l,3}\right)$

17:  $X_{\text{de}}^{l}=S_{\text{de}}^{l,3}$

18: **end for**

19: $X_{\text{pred}}=\text{Linear}\left(X_{\text{de}}^{M}\right)+T_{\text{de}}^{M}$

20: **return** *X*_pred_

## Experiment

### Datasets

**Fig 6:**
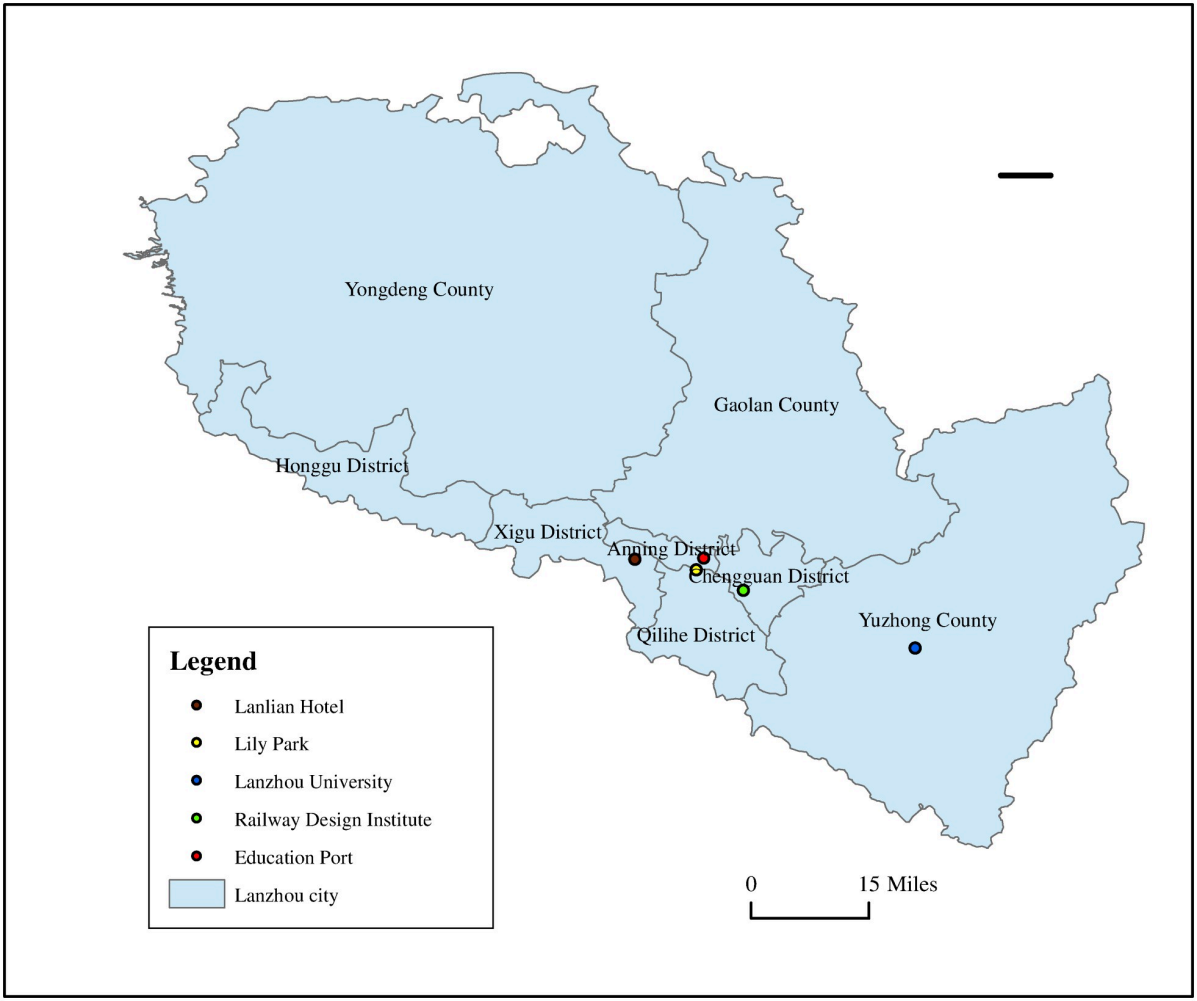
The locations of five selected typical districts in Lanzhou.

Lanzhou City, the capital of Gansu Province, is an important transportation hub in northwest China. It is also one of the important node cities of the Silk Road Economic Belt. Lanzhou has jurisdiction over five districts and three counties. Lanzhou City has a total area of 13,100 square kilometers and a resident population of 4,384,300. Lanzhou is also an important national industrial base for petrochemical, biopharmaceutical and equipment manufacturing. With the continuous and rapid development of social economy and the rapid increase of energy consumption, Lanzhou City is facing more and more environmental pressure, especially the air pollution problem in the urban area is becoming more and more prominent. In this paper, the study was conducted based on the hourly datasets collected from the four districts (Chengguan, Qilihe, Anning, and Xigu) and one county (Yuzhong). The location of air quality monitoring stations in Lanzhou is shown in Fig 6. The AQI from January 1, 2019, to May 31, 2022, was drawn from the web https://www.epmap.org, including O_3_, PM_10_, PM_2.5_, NO_2_, CO, SO_2_, AQI indicators. Table 2 presents the basic statistical characteristics of air quality for five datasets. The missing data are processed by the linear interpolation method. The data is normalized by Z-Score method.

**Table 2:** The basic statistical characteristics of five datasets.

Dataset	Feature	Mean	Std	Min	Max	First Quartile	Median	Third Quartile
Chengguan	SO_2_	17.177	13.820	3.000	268.000	7.000	12.000	23.000
	NO_2_	56.741	29.211	6.000	216.000	33.000	53.000	74.000
	CO	1.164	0.975	0.000	12.400	0.500	0.800	1.500
	O3	50.989	39.958	0.000	271.000	15.000	45.000	77.000
	PM_10_	94.556	159.896	0.000	4379.000	48.000	74.000	107.000
	PM_2.5_	36.334	31.849	0.000	721.000	20.000	31.000	44.000
	AQI	71.485	47.389	0.000	500.000	49.000	64.000	81.000
Qilihe	SO_2_	16.591	10.823	1.000	135.000	10.000	13.000	20.000
	NO_2_	48.560	29.784	1.000	185.000	22.000	44.000	69.000
	CO	0.794	0.584	0.000	5.600	0.400	0.600	1.000
	O_3_	52.505	46.454	0.000	268.000	6.000	50.000	85.000
	PM_10_	97.497	180.939	0.000	4128.000	44.000	69.000	102.000
	PM_2.5_	40.807	41.391	0.000	682.000	22.000	32.000	47.000
	AQI	74.879	60.574	15.000	500.000	47.000	62.000	83.000
Anning	SO_2_	14.785	11.852	0.000	161.000	7.000	11.000	18.000
	NO_2_	43.550	27.990	1.000	204.000	20.000	38.000	64.000
	CO	0.893	0.627	0.000	7.500	0.500	0.700	1.100
	O_3_	60.692	45.854	1.000	298.000	18.000	58.000	90.000
	PM_10_	101.835	179.403	0.000	4448.000	51.000	77.000	113.000
	PM_2.5_	35.409	32.503	0.000	699.000	21.000	30.000	41.000
	AQI	75.042	52.282	0.000	500.000	52.000	66.000	84.000
Xigu	SO_2_	17.144	17.368	2.000	191.000	6.000	11.000	21.000
	NO_2_	44.715	26.907	2.000	270.000	22.000	40.000	61.000
	CO	0.847	0.513	0.000	5.400	0.500	0.700	1.100
	O_3_	62.456	50.160	0.000	536.000	18.000	57.000	92.000
	PM_10_	94.736	149.341	0.000	4400.000	49.000	75.000	107.000
	PM_2.5_	44.992	38.738	0.000	885.000	26.000	37.000	53.000
	AQI	75.804	49.900	0.000	500.000	51.000	66.000	87.000
Yuzhong	SO_2_	8.791	8.810	1.000	185.000	4.000	5.000	11.000
	NO_2_	20.971	14.397	1.000	203.000	12.000	17.000	27.000
	CO	0.774	0.496	0.000	10.000	0.500	0.600	0.900
	O_3_	85.929	30.179	2.000	303.000	64.000	84.000	105.000
	PM_10_	74.858	140.363	0.000	3774.000	38.000	56.000	83.000
	PM_2.5_	27.383	25.392	0.000	607.000	16.000	23.000	34.000
	AQI	60.578	44.959	0.000	500.000	41.000	54.000	68.000

### Evaluation metrics

In order to evaluate the performance of the model, three metrics are used to evaluate the model, namely mean squared error (MSE), mean absolute error (MAE) and root mean square error (RMSE).

$$\begin{array}{c} \text{MSE}=\frac{1}{n}\sum_{i=1}^{n}{\left(y_{i}-{\hat{y}}_{i}\right)}^{2}, \end{array}$$ (21)

$$\begin{array}{c} \text{MAE}=\frac{1}{n}\sum_{i=1}^{n}\left|y_{i}-{\hat{y}}_{i}\right|, \end{array}$$ (22)

$$\begin{array}{c} \text{RMSE}=\sqrt{\frac{1}{n}\sum_{i=1}^{n}{\left(y_{i}-{\hat{y}}_{i}\right)}^{2}}, \end{array}$$ (23)

where *y*_*i*_ is the actual value of the AQI, ${\hat{y}}_{i}$ is the predicted value, and *n* is the number of samples. The lower value of the MSE, MAE and RMSE, the better performance of the model.

Three improvement percentage metrics were also used to present the accuracy improvement of the proposed model compared to the baseline model.

$$\begin{array}{c} {\text{P}}_{\text{MSE}}=\frac{{\text{MSE}}_{\text{base}}-{\text{MSE}}_{\text{prop}}}{{\text{MSE}}_{\text{base}}}\times 100\% \end{array}$$ (24)

$$\begin{array}{c} {\text{P}}_{\text{MAE}}=\frac{{\text{MAE}}_{\text{base}}-{\text{MAE}}_{\text{prop}}}{{\text{MAE}}_{\text{base}}}\times 100\%, \end{array}$$ (25)

$$\begin{array}{c} {\text{P}}_{\text{RMSE}}=\frac{{\text{RMSE}}_{\text{base}}-{\text{RMSE}}_{\text{prop}}}{{\text{RMSE}}_{\text{base}}}\times 100\%, \end{array}$$ (26)

where the subscript “prop”in Eqs 24–26 refers to the proposed model, and the subscript “base”refers to the baseline model.

### Baselines

To evaluate the prediction performance of the proposed model, we use five baselines for comparison. (1) LSTM [26]: The long short-term memory network (LSTM) is the most commonly used method for time series forecasting problems. (2) Transformer [29]: Transformer is a classical model of NLP proposed by Google’s team in 2017 based on the Attention mechanism. (3) Informer [33]: Informer is a long sequence time-series forecasting based on improved Transformer model. (4) Autoformer [34]: Autoformer model is a prediction model based on deep decomposition architecture and autocorrelation mechanism for problems in long time series prediction. (5) TCCT [35]: The tightly-coupled convolutional Transformer (TCCT) is a novel forecasting model that combines Transformer and CNN tightly.

### Experimental settings

The datasets are divided into training, validation and test set by 7:2:1. During the training process, all methods are optimized with Adam optimizer with the initial learning rate of 0.0001, Dropout is 0.05 and the loss function is the MSE loss. The total number of epochs is 20. Batch size is set as 48. The experiments are implemented in PyTorch and conducted on a single GeForce RTX 2080 Ti 11 GB GPU.

### Results

The MSE, MAE, RMSE, and the corresponding improvement percentages of the proposed model and baselines are provided in Tables 3 and 4, respectively. The following conclusions can be seen from the Table 1: (1) Compared with the LSTM, Informer, Transformer and Autoformer, the models including the TCCT and our EnAutoformer exhibit better prediction performance in all districts. The major difference between two models (TCCT and EnAutoformer) and the previous four models is the use of CSPDenseNet strategy, which utilizes the cross-stage feature fusion mechanism and integrates the feature maps of each phase of the network. The results show that the model with the CSPNet significantly outperforms the other models in terms of accuracy. (2) Compared with the TCCT, the MSE, MAE and RMSE reduction realized by the proposed model for all datasets. Although the proposed model achieved a relatively small reduction in MAE of 0.80%, 2.16% and 3.24% for the three datasets (Chengguan,Xigu,Yuzhong) respectively, other metrics suggest that our EnAutoformer significantly outperforms the TCCT.

**Table 3:** Performance comparison of the proposed model and baselines.

Dataset	Metric	LSTM	Informer	Transformer	Autoformer	TCCT	EnAutoformer
Chengguan	MSE	2.656	2.284	2.714	2.583	1.194	0.415
	MAE	0.793	0.648	0.811	0.905	0.501	0.497
	RMSE	1.630	1.511	1.647	1.607	1.092	0.644
Qilihe	MSE	0.850	1.042	0.971	1.096	0.765	0.109
	MAE	0.492	0.523	0.503	0.579	0.441	0.267
	RMSE	0.922	1.020	0.985	1.049	0.875	0.330
Anning	MSE	1.385	1.275	1.111	1.474	1.098	0.349
	MAE	0.628	0.563	0.529	0.709	0.577	0.468
	RMSE	1.177	1.129	1.054	1.214	1.048	0.590
Xigu	MSE	2.250	2.119	2.393	2.211	1.127	0.514
	MAE	0.773	0.759	0.745	0.691	0.556	0.544
	RMSE	1.500	1.455	1.547	1.487	1.061	0.717
Yuzhong	MSE	2.242	1.977	1.881	2.126	1.192	0.547
	MAE	0.713	0.654	0.670	0.739	0.556	0.538
	RMSE	1.497	1.406	1.371	1.458	1.091	0.739

**Table 4:** Improvement percentages of the proposed model and baselines.

Dataset	Metric	LSTM	Informer	Transformer	Autoformer	TCCT
Chengguan	P_MSE_	84.38%	81.83%	84.71%	83.93%	65.24%
	P_MAE_	37.33%	23.30%	38.72%	45.08%	0.80%
	P_RMSE_	60.49%	57.38%	60.90%	59.93%	41.03%
Qilihe	P_MSE_	87.18%	89.54%	88.77%	90.05%	85.75%
	P_MAE_	45.73%	48.95%	46.92%	53.89%	39.46%
	P_RMSE_	64.21%	67.65%	66.50%	68.54%	62.29%
Anning	P_MSE_	74.80%	72.63%	68.59%	76.32%	68.21%
	P_MAE_	25.48%	16.87%	11.53%	33.99%	18.89%
	P_RMSE_	49.87%	47.74%	44.02%	51.40%	43.70%
Xigu	P_MSE_	77.16%	75.74%	78.52%	76.75%	54.39%
	P_MAE_	29.62%	28.33%	26.98%	21.27%	2.16%
	P_RMSE_	52.20%	50.72%	53.65%	51.78%	32.42%
Yuzhong	P_MSE_	75.60%	72.33%	70.92%	74.27%	54.11%
	P_MAE_	24.54%	17.74%	19.70%	27.20%	3.24%
	P_RMSE_	50.63%	47.44%	46.10%	49.31%	32.26%

We also analyze the performance of all models for long-term predictions. Table 5 shows the performance comparison of different models under different prediction horizons. In Table 5, 12h,24h and 36h represent the 12-hour, 24-hour and 36-hour prediction horizons, respectively. It can be observed that the accuracy of short-term AQI prediction is higher than that of long-term prediction. As the prediction horizons increases, the prediction performance of all models gradually decreases. Compared with baselines, the all evaluation metrics of our proposed model are the smallest among others. These results indicate that, among the long-term prediction, the EnAutoformer model yields the most accurate results and exhibits an efficient prediction performance.

**Table 5:** Performance comparison of different prediction horizons.

Dataset	Models	12h			24h			36h		
		MSE	MAE	RMSE	MSE	MAE	RMSE	MSE	MAE	RMSE
Chengguan	LSTM	2.100	0.691	1.449	2.656	0.793	1.630	2.666	0.795	1.633
	Informer	2.243	0.650	1.497	2.284	0.648	1.511	2.446	0.727	1.564
	Transformer	2.007	0.598	1.416	2.714	0.811	1.647	2.440	0.739	1.562
	Autoformer	2.299	0.771	1.516	2.583	0.905	1.607	2.639	0.878	1.624
	TCCT	1.159	0.483	1.076	1.194	0.501	1.092	1.228	0.503	1.108
	EnAutoformer	0.390	0.476	0.625	0.415	0.497	0.644	0.558	0.591	0.747
Qilihe	LSTM	0.368	0.389	0.607	0.850	0.492	0.922	0.855	0.492	0.925
	Informer	0.971	0.491	0.985	1.042	0.523	1.020	1.081	0.513	1.040
	Transformer	0.897	0.448	0.947	0.971	0.503	0.985	0.971	0.460	0.985
	Autoformer	1.061	0.534	1.030	1.096	0.579	1.049	1.081	0.615	1.039
	TCCT	0.606	0.363	0.779	0.765	0.441	0.875	0.798	0.476	0.893
	EnAutoformer	0.105	0.254	0.324	0.109	0.267	0.330	0.252	0.402	0.502
Anning	LSTM	1.385	0.628	1.177	1.385	0.628	1.177	1.397	0.630	1.182
	Informer	0.972	0.453	0.986	1.275	0.563	1.129	1.157	0.538	1.075
	Transformer	0.969	0.464	0.984	1.111	0.529	1.054	1.212	0.595	1.101
	Autoformer	1.260	0.641	1.122	1.474	0.709	1.214	1.465	0.720	1.210
	TCCT	0.849	0.515	0.921	1.098	0.577	1.048	1.181	0.561	1.087
	EnAutoformer	0.322	0.452	0.567	0.349	0.468	0.590	0.660	0.662	0.812
Xigu	LSTM	2.242	0.773	1.497	2.250	0.773	1.500	2.258	0.775	1.503
	Informer	1.960	0.634	1.400	2.119	0.759	1.455	2.057	0.686	1.434
	Transformer	1.755	0.576	1.324	2.393	0.745	1.547	2.361	0.829	1.536
	Autoformer	2.051	0.779	1.432	2.211	0.691	1.487	2.439	0.892	1.562
	TCCT	1.002	0.548	1.001	1.127	0.556	1.061	1.220	0.592	1.104
	EnAutoformer	0.453	0.539	0.673	0.514	0.544	0.717	0.541	0.574	0.735
Yuzhong	LSTM	2.233	0.712	1.494	2.242	0.713	1.497	2.249	0.714	1.500
	Informer	1.660	0.569	1.288	1.977	0.654	1.046	2.034	0.670	1.426
	Transformer	1.635	0.562	1.279	1.881	0.670	1.371	2.314	0.819	1.521
	Autoformer	1.939	0.710	1.392	2.126	0.739	1.458	2.393	0.836	1.547
	TCCT	1.017	0.478	1.008	1.192	0.556	1.091	1.110	0.568	1.053
	EnAutoformer	0.529	0.517	0.727	0.547	0.538	0.739	0.654	0.614	0.809

## Conclusions

This study aims at enhancing the prediction performance of air quality by using deep learning. In this paper, we proposed a novel encoder-decoder model named as EnAutoformer to improve the AQI prediction. The encoder layer consisting of several identical blocks stacked together, including the CSP_ECC and FeedForward blocks. The decoder layer consists of several decoder blocks including CSP_ECC, SeriesDecomp, FeedForward and DCC block. The CSP_ECC block, which was based on cross-stage feature fusion mechanism of CSPDenseNet and an enhanced cross-correlation mechanism, is not only able to extract the temporal dependencies in time series, but also to improve the computational efficiency. The time series decomposition was employed to further obtain the intrinsic features of time series including seasonality and trend. The DCC was designed for extracting long-term dependence of AQI. The effective integration of these techniques enhanced the predictive performance of the proposed model. Various metrics like MSE, MAE and RMSE were used for evaluating the proposed model and baselines. Experimental results on real-world show that our EnAutoformer model exhibited the best performance in all datasets and outperformed the existing baselines.

According to the conclusions of this study, future work can concentrate on the following aspects:(1) A shortcoming of the model is that it takes one monitoring location in each district in Lanzhou. The external influencing factors should be added to build prediction models, such as meteorological factors, topography, and geomorphology, etc. Datasets containing rich information can be used in future.(2) Many methods are available to improve the performance and efficiency of deep learning-based predictive models. These methods mainly include data preprocessing [20], deep learning model improvement [32, 33, 35], improvement of neural networks based on optimization algorithms [38, 39], and other hybrid models [25, 26, 28]. We continue to experiment with various methods to improve model prediction accuracy and efficiency, such as feature selection, multi-objective optimization techniques, model improvement, etc.

## Supporting information

S1 File (RAR)
